# Can High Altitude Influence Cytokines and Sleep?

**DOI:** 10.1155/2013/279365

**Published:** 2013-04-09

**Authors:** Valdir de Aquino Lemos, Ronaldo Vagner Thomatieli dos Santos, Fabio Santos Lira, Bruno Rodrigues, Sergio Tufik, Marco Tulio de Mello

**Affiliations:** ^1^Departamento de Psicobiologia, Campus São Paulo, UNIFESP, Rua Botucatu 862, Vila Clementino, São Paulo, SP, Brazil; ^2^Centro de Estudos em Psicobiologia e Exercício, (CEPE), São Paulo, SP, Brazil; ^3^Departamento de Biociências, Campus da Baixada Santista, UNIFESP, Avenida Almirante Saldanha da Gama, 89, Ponta da Praia, 11030-400 Santos, SP, Brazil; ^4^Centro de Estudos em Psicobiologia e Exercício, UNIFESP, Rua Professor Francisco de Castro 04020-050, 93, Vila Clementino, São Paulo, SP, Brazil; ^5^Laboratório do Movimento Humano, Universidade São Judas Tadeu, São Paulo, SP, Brazil; ^6^Unidade de Hipertensão, Instituto do Coração (InCor), Faculdade de Medicina da Universidade de São Paulo, São Paulo, SP, Brazil

## Abstract

The number of persons who relocate to regions of high altitude for work, pleasure, sport, or residence increases every year. It is known that the reduced supply of oxygen (O_2_) induced by acute or chronic increases in altitude stimulates the body to adapt to new metabolic challenges imposed by hypoxia. Sleep can suffer partial fragmentation because of the exposure to high altitudes, and these changes have been described as one of the responsible factors for the many consequences at high altitudes. We conducted a review of the literature during the period from 1987 to 2012. This work explored the relationships among inflammation, hypoxia and sleep in the period of adaptation and examined a novel mechanism that might explain the harmful effects of altitude on sleep, involving increased Interleukin-1 beta (IL-1**β**), Interleukin-6 (IL-6), and tumor necrosis factor-alpha (TNF-**α**) production from several tissues and cells, such as leukocytes and cells from skeletal muscle and brain.

## 1. Background

In recent years, the interest in activities carried out at high altitudes has grown. Millions of people travel to regions of high altitudes (i.e., above 2500 m) for tourism, sport, work, or permanent residence. However, living in high altitudes can lead to hypoxia. The effects of exposure to hypobaric hypoxia, which is present at high altitude, are dependent on the length of time spent at high altitude and the altitude reached.rt Because O_2_ is required for the maintenance of vital functions, blood oxygenation can affect several physiological functions. Exposure to hypobaric hypoxia can result in extreme conditions, such as acute mountain sickness (AMS), high altitude pulmonary edema (HAPE), and high altitude cerebral edema (HACE), as well as other conditions, such as headache, nausea, vomiting, and gastrointestinal alterations [1–5].

 Alterations in cardiovascular and respiratory functions promoted by altitude have been previously described. More recently, attention has focused on neurobiological functions, including sleep, cognition, and humor [6, 7]. Thus, this review discusses the effects of hypoxia stimulated by high altitude on sleep, with an emphasis on neuroimmunoendocrine interactions.

## 2. Methods

For this study, we conducted a systematic and integrative review of the literature, using source articles indexed by the ISI database, PubMED and MEDLINE by searching for books that addressed specific aspects related to altitude/hypoxia, cytokines, and sleep during the period from 1987 to 2012.

The keywords searched were “*cytokines and hypoxia,*” “*cytokines and altitude,*” “*inflammation and hypoxia,*” “*inflammation and altitude,*” “*sleep and hypoxia,*” “*sleep and altitude,*” “*sleep and cytokines,*” and “*sleep and inflammation*.” These descriptors were used in a Boolean-specific basis to obtain various arrangements thought to maximize both the coverage and quality of the search. No restrictions were made regarding age or gender. 

## 3. Altitude

The principal characteristic of exposure to high altitudes is the fact that there is an inverse correlation between altitude and the partial pressure of O_2_. Therefore, at high altitudes, the body tries to adapt by generating many responses, including changes in skeletal muscle and in the endocrine and nervous systems [8].

Although the barometric pressure decreases with increasing altitude, the gas composition does not change until above the 1200 m level. Although the percentage of ambient oxygen is maintained at 20.93%, the increase in altitude decreases the O_2_ partial pressure in expired air. This decrease promotes a partial impairment in the support of O_2_, resulting in less oxygen transported by hemoglobin and consequently less O_2_ available for tissues. In fact, all tissues that need O_2_ for energy production are affected by hypoxia, and each tissue response depends on several factors, including the O_2_ demand by the tissue, the time of exposure, and the individual's characteristics [9].

The classical response induced by high altitude includes respiratory and cardiovascular changes that are initiated within minutes after the person reaches the altitude [10]. In fact, there is an inverse correlation between increases in altitude and hemoglobin saturation. In addition, the number of hemoglobin molecules begins to increase, even at altitudes as low as 500 m. At the same time, alterations in hyperventilation occur at rest and during acute physical exercise. The heart rate increases in a manner similar to the increase seen in cardiac output, which attempts to compensate by decreasing the partial pressure of carbon dioxide in the arterial blood (PaCO_2_); however, these alterations are not sufficient to affect the oxygen consumption (VO_2_) decrease and aerobic energy production. As a result, remaining at high altitudes might result in fatigue and a significant decrease in the capacity to work and physically perform, especially aerobic and endurance exercise. In addition, it is possible to have an increase in blood pressure due to an increase of norepinephrine levels because of the impact of stimulated activities of rest and exercise [11, 12].

High altitude (above 3000 m) is a powerful stressor. Being at these altitudes can modify metabolic and physiological functions, and the body then tries to reestablish the homeostasis that was altered by hypoxia [13]. Several studies have shown that acute or chronic exposure to altitudes between 2500 and 5000 m results in sympathoadrenal responses that are exacerbated by metabolic alterations to other systems [13], including the immune system [12, 14].

Under these conditions, it is possible to produce a rapid adrenaline hormonal response and a transient increase in plasma cortisol concentrations [15, 16].

Altitude-induced hypoxia can also stimulate the release of other hormones involved in the recovery of homeostasis. One of those hormones is erythropoietin (EPO). Humans exhibit increased EPO concentrations two hours after exposure to high altitudes [17]. EPO is fundamentally important to the organization of the physiological response to altitude and can modulate the expression of many proteins. Increases in EPO and hemoglobin are essential for acclimatization and the maintenance of the O_2_ supply to tissues.

It has been demonstrated that acute exposure to elevated altitudes can result in changes to several immunological parameters [12, 18]. Hypoxia for even a few hours is sufficient to induce significant changes in neutrophil and lymphocyte numbers, which are mainly characterized by reductions in cluster of differentiation (CD), cell numbers, and cellular proliferation [19]. Several studies have shown that acute hypoxia results in an increase in natural killer cells (NK cells) numbers and activity [20].

Studies have shown that lymphocytes and phagocytes present some signs of adaptation if the hypobaric stimulus is chronic, due to alterations in the production and release of substances such as cytokines and antibodies [21]. Other studies have shown that immunity mediated by T lymphocytes can be stopped by exposure to elevated altitudes [12, 22]. Facco et al. [21] confirmed that exposure to elevated altitudes can alter the number and cellular function and suggested that new studies be carried out to evaluate the expression of cytokines by T lymphocytes, particularly to determine the maintenance of the T helper cells (Th1/Th2) response. 

It was suggested that remaining at an altitude of 4000 m above sea level was associated with increased plasma concentrations of IL-6 and Interleukin-1 receptor antagonist (IL-1ra). Furthermore, C-reactive protein (CRP) increases are associated with the development of pulmonary edema [23]. Numerous stressful events are associated with increases in cytokine release and disturbances in the pro/anti-inflammatory cytokine ratio [24]. Hypoxia alone seems to have a decisive role; however, the mechanisms responsible for the induction of cytokines under hypoxic conditions are not clear. Exposure to elevated attitudes can cause cellular damage due to increased oxidative stress and altered cytokine release; in turn, these cytokines participate in the recovery from cellular damage [25, 26]. 

## 4. Altitude and Inflammation

The exposure to hypoxia promotes several transcription factors, including nuclear factor-*κ*B (NF-*κ*B), which plays a central role in stimulating the proinflammatory cytokines TNF-*α* and IL-6 [27]. Similarly, several studies with rodents and humans have shown that effects-induced hypoxia can cause inflammation, including increase in transvascular leakage and oxidative stress with increased NF-*κ*B expression in lungs followed by significant increase in proinflammatory cytokines IL-1, IL–6, and TNF-*α* [28–30].

A decrease in plasma cytokine concentration or the treatment with appropriate antagonists promotes partial reversion of the symptoms and illnesses, including cardiovascular disease, obesity, insulin resistance, and depression [31, 32]. Therefore, we suggest that sleep disturbances due to high altitudes could also be caused by increases in proinflammatory cytokines from several cells, such as skeletal muscle and immune cells, in association with the capillary leakage or repeated wakening aspects of AMS, which usually occur concurrently with the hyperopic phase of periodic breathing.

Hojman et al. [33] observed that augments the acute inflammatory effect in humans. In this study, the authors demonstrated that when EPO was given prior to a bolus injection of endotoxin, the levels of TNF-*α* and IL-6 were enhanced by 5- and 40-fold, respectively, whereas the endotoxin-induced increase in Interleukin-10 (IL-10) was not influenced by EPO. This interaction between EPO and inflammation may corroborate with sleep disruptions found at high altitudes.

However, Hojman et al. [34] used animal experiments to show that when EPO was expressed at supraphysiological levels, there were substantial metabolic effects, including protection against diet-induced obesity and normalization of glucose sensitivity, associated with a shift towards increased fat metabolism in the muscles.

Unfortunately, only limited information from well-controlled laboratory and field studies is available on this topic. Relatively, little is known about the influence of altitude on the interaction of cytokines and sleep. The significant effects (pro- and anti-inflammatory) of EPO in acute and chronic high altitudes should be investigated further. Thus, the sleep complains due to high altitudes could also be caused by increases in proinflammatory cytokines from several cells, such as skeletal muscle and immune cells, in association with the capillary leakage or repeated wakening aspects of AMS, which usually occur concurrently with the hyperopic phase of periodic breathing. This interaction between EPO and inflammation may corroborate with sleep disruptions found at high altitudes.

## 5. Cytokines

Cytokines are proteins produced and released by different cells, for example: leukocytes, muscle cells, and neurons. These proteins can act in a pleiotropic way or in synergy with other substances and can modulate the production of other cytokines [35]. Cytokines function in the regulation of metabolism by influencing hormone secretion, regulating the TH1/TH2 immune responses, and inducing inflammatory responses; in the nervous system, they influence complex neuronal actions and modulate thermoregulation, food intake, and neurobiological patterns [35, 36] during sleep.

 Interleukin-1 (IL-1) increased in plasma concentration may cause fever, sickness behavior, increased heart rate, increased blood flow in many vascular beds, and increased sympathetic tone; changes in carbohydrate, fat, and protein metabolism also occur [24, 35]. The effects of IL-1 can be reversed by treatment with IL-1ra, an antagonist of IL-1, which functions to prevent IL-1 binding to its specific receptors [35].

 The TNF-*α* is mainly produced by macrophages and neutrophils, but other cells, such as lymphocytes, NK cells, endothelial cells and neural cells, might also have the capacity to produce it [24]. TNF-*α* production occurs in response to a wide variety of stimuli, including infections and stimulation by other cytokines or mitogens [37]. TNF-*α* is a potent pleiotropic cytokine due to its ability to activate multiple signal transduction pathways and induce or suppress the expression of a number of genes. In addition, it has potent endogenous pyrogenic properties and may promote changes in the body's physiological temperature [38]. Moreover, tissues that present marked cachexia show high TNF-*α* activity, as observed in catabolic conditions, such as cancer and systemic inflammatory diseases [24]. 

 The Interleukin-6 (IL-6) plays a significant role in regulating the pro-inflammatory response [24]. However, due to its capacity to stimulate the hypothalamus-pituitary-adrenal axis to produce cortisol and anti-inflammatory cytokines, such as interleukin-4 (IL-4) and it also has anti-inflammatory properties [24].

 The Interleukin-10 (IL-10) has multiple biological activities and affects many different cell types. These include monocytes/macrophages, T cells, B cells, NK cells, neutrophils, endothelial cells, and peripheral blood mononuclear cells (PBMCs). IL-10 also acts in the regulation of inflammation because it is produced by adipose and muscle tissues, which are important to the pro/anti-inflammatory ratio in conditions such as physical exercise, obesity, and inflammatory diseases [39, 40].

**Figure 1:**
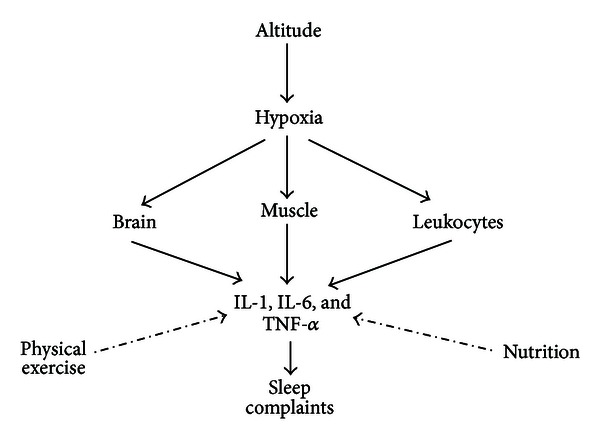
Solid line indicates stimulation; dotted line indicates inhibition.

 Cytokines can penetrate the blood-brain barrier (BBB) and act indirectly on the brain by stimulating the production of chemical second messengers that carry information to targets such as NF-*κ*B and adenosine [41, 42] as shown in Figure 1. The hypothesis that cytokines could influence the functions of the nervous system (NS) is based on observations that treatment with cytokines, such as Interferon-*γ* (INF-*γ*), promotes neuroendocrine alterations, and other studies show that there are receptors for these cytokines in many areas of the brain [38, 43, 44]. Additional studies have shown that an increase in proinflammatory cytokine concentrations promotes a decrease in the transendothelial electrical resistance and an increase in the permeability of the BBB [45]. Finally, it is possible that cytokines can be produced within the brain itself in response to neuronal activity [35].

 More recently, several studies have shown the existence of an afferent neural pathway by which inflammation in the peritoneal cavity might influence the brain [46]. Subdiaphragmatic transection of the vagus produces reduction of fever, poor sleep, nocturnal excretion of norepinephrine, and hypothalamic production of IL-1 induced by lipopolysaccharides (LPS) in the peritoneal cavity [47], thereby validating this hypothesis. These alterations are not due to a reduction in the circulating levels of cytokines or to the attenuation of the inflammatory response induced by lipopolysaccharide (LPS) but rather to a defective translation of cytokines in the brain [48].

 High altitudes are potent stressors known to alter physiological and metabolic functions in the search for mechanisms to try to restore homeostasis by hypoxia disbalance. The acute or chronic exposure to altitudes between 2500 and 5000 m stimulates in a response sympathoadrenal leading to numerous other metabolic changes that, in turn, could affect several physiological systems including the production of cytokines and worsen the quality of sleep [49–51]. 

 Currently, a strong relationship between sleep and immune process has been shown. The proinflammatory cytokines, including IL-1, IL-6, and TNF-*α*, are known as sleep-regulatory cytokines. However, sleep-promoting properties are also possessed by several other immune and proinflammatory cellular classes. Many studies reporting these relationships are focused on the perspective of low-grade inflammation associated with significant sleep alterations and on the perspective of immune dysregulation associated with several primary sleep disorders [52]. 

## 6. Altitude and Sleep

Sleep is a functional state that includes a complex combination of physiological and behavioral processes. It has some characteristic manifestations, such as a cyclic pattern, relative immobility, and an increase in the response threshold to external stimuli [53]. Sleep is very important, as it is evident from studies of acute or chronic sleep deprivation and sleep disorders; these impairments promote several alterations, including a marked increase in the production of stress hormones, including catecholamines and cortisol, a reduction in cognitive capacity, and a reduction in the state of alertness, among others [54].

 Sleep can be divided into two phases: the nonrapid eye movement (nREM) phase, in which the electroencephalogram (EEG) records a synchronized tracing, and the rapid eye movement (REM) sleep phase, in which the electroencephalogram records signals similar to those in the wake period that are associated with the rapid eye movements [55, 56].

 Two hypotheses attempt to explain the mechanisms involved in sleep regulation, and it is possible that these hypotheses are not mutually exclusive and could happen simultaneously. One hypothesis describes the role of circadian rhythms, while the other is related to the homeostatic effects of sleep [55].

 The biochemical mechanisms that control sleep are very complex because sleep modulation is dependent on several factors, including carbon dioxide (CO_2_) concentrations, as well as potassium, free radical, nitric oxide, hormone, and adenosine levels [57]. Proinflammatory cytokines play an important role in sleep regulation [58]. Some cytokines have an antisomnogenic action by decreasing prosomnogenic cytokine production, while others cytokines have the opposite effect [59]. 

 Most of the existing studies on sleep and altitude were carried out in the field. There have also been studies carried out in normobaric hypoxic rooms that simulate conditions of high altitude [60]. High altitude has frequently been associated with sensations of suffocation when awakening from sleep. In fact, several studies showed that hypoxia directly acts on the architecture and quality of sleep in humans and rodents; these effects include increases in Stage I, decreases in REM sleep, lesions in cerebral regions that control sleep, and increases in the sensations of sleep deprivation and sleep fragmentation [61–63].

In fact, around 60% of persons subjected to altitudes of 3500 m or higher experience various sleep complaints. Recurring wakefulness is the most common characteristic due to the decreased O_2_ saturation, which leads to sleep fragmentation [45, 64, 65]. In addition, hypoxia can cause poor sleep quality due to slight reductions in delta sleep, relative reductions in REM sleep, and agitation during the night [63]; however, overall total sleep time (TST) is not reduced. Therefore, the reduced subjective sleep quality is due to a higher arousal frequency. Despite previous studies suggesting that the impairment of sleep persists even after a season of acclimatization [64, 65], partial recovery of the damage during sleep can occur after spending some days at high altitude [26]. This finding has been shown in animal studies in which several days were spent in hypoxic conditions but not after a sudden ascent.

## 7. Altitude, Sleep, and Cytokines

To date, the effects of altitude on the architecture and quality of sleep are not well known [66]. Studies in rodents and humans suggest that prolonged exposure to hypoxia can alter circadian rhythms by reducing the amplitude of circadian oscillations and by possibly leading to changes in several variables, such as activity, hunger, metabolic rate, and the dark and light cycle [60, 67, 68]. The modification of melatonin and neurotransmitter release, metabolism in peripheral tissues, and modulation of several hormones and cytokines that participate in sleep regulation and gene expression responsible for the functions of the biological clock are also affected [69–71]. In part, this alteration on the sleep leads to upregulation of proinflammatory cytokines in response at high altitude. 

The relationship between sleep and cytokines was first established through observations that sleep deprivation increases INF-*γ* production. To date, the roles of several growth factors, including epidermal growth factor, fibroblast growth factor, nerve growth factor, brain-derived neurotrophic factor, granulocyte-macrophage colony-stimulating factor and insulin-like growth factor-1 (IGF-1), have also been investigated for their roles in sleep modulation [59]. However, this review focuses on the pro- and anti-inflammatory cytokines IL-1*β*, IL-6, IL-10, and TNF-*α*. 

## 8. Hypoxia, Physical Exercise, Cytokines and Sleep

In relation to physical exercise in hypoxia, few and contradictory studies evaluated the effect of exercise on condition of hypoxia on the production of cytokines [72]. The exercise performed under hypoxic conditions/high altitude represents an additional stress condition in relation to the exercise performed at sea level [73]. Even when the exercise intensity is relative, that is, taking into account that the maximum VO_2_ and performance decreases as the altitude increases [73, 74]. So many factors should be taken into account when discussing the interaction hypoxia and cytokines. The increase in altitude or the extent of hypoxia is a primary factor that influences the level of variation in physiological and biochemical parameters which can modulate the immune response mediated by exercise [12]. Collectively, analyzing the results of the previously published works, one can speculate that there is a threshold elevation that should be followed to achieve the benefits associated with living or training at altitude with the least possible damage [12].

 The concentration of cytokines, notably IL-6 and inflammatory markers such as the acute phase proteins CRP has its increased concentrations in response to a session with moderate exercise intensity of 50% VO_2__*Max*⁡_ at an altitude of 4300 m over the same exercise at sea level [75]. However, in this study, the authors evaluated the effects of varying intensities of exercise in normoxic and hypoxic environments at equivalently 3100 m on immune regulation and metabolic responses and showed that during prolonged physical exercise at 40 and 60% of VO_2__*Max*⁡_ this doesnot seem to dramatically alter the response of the selected immune system including IL-1 or TNF-*α* and metabolic markers. Exercise training that uses acute hypoxic environments does not adversely affect immune regulation system status and may be beneficial for those individuals looking to increase endurance performance [76]. 

 One way to partially reverse the effects of hypoxia on sleep patterns can be by performing moderate exercise, taking into account that in normoxic condition physical exercise beside improving sleep also modulates the memory, attention, and mood state [77].

Physical exercise has been considered as the best strategy to prevent and treat chronic inflammatory diseases of low grade [78, 79], such as those generated by sleep disorders. Regular physical training is able to increase the production of anti-inflammatory cytokines and decrease the concentrations of circulating proinflammatory cytokines and can improve the quality of sleep. 

## 9. Conclusions

The relationships among inflammation, hypoxia, and sleep are discussed in the present study; we conclude that hypoxia induced by elevated altitudes in the adaptation period results in a disturbance in the balance of homeostasis and affects several physiological systems. Consequently, severe changes in sleep architecture and sleep quality may occur. These changes might be mediated by increases in plasma concentrations of IL-1, IL-6, and TNF-*α* and possibly through the stimulation of EPO.
